# Shengui Sansheng San Ameliorates Cerebral Energy Deficiency via Citrate Cycle After Ischemic Stroke

**DOI:** 10.3389/fphar.2019.00386

**Published:** 2019-04-23

**Authors:** Cheng Luo, Xiqing Bian, Qian Zhang, Zhenyan Xia, Bowen Liu, Qi Chen, Chienchih Ke, Jian-Lin Wu, Yonghua Zhao

**Affiliations:** ^1^State Key Laboratory of Quality Research in Chinese Medicine, Faculty of Chinese Medicine, Macau University of Science and Technology, Macau, China; ^2^Department of Biotherapy, Shenzhen Luohu People’s Hospital, Shenzhen, China; ^3^Department of Medical Imaging and Radiological Sciences, Kaohsiung Medical University, Kaohsiung, Taiwan; ^4^Biomedical Imaging Research Center, National Yang-Ming University, Taipei, Taiwan; ^5^State Key Laboratory of Quality Research in Chinese Medicine, Institute of Chinese Medical Sciences, University of Macau, Macau, China

**Keywords:** cerebral energy deficiency, citrate cycle, DIAAA derivatization-UHPLC-Q-TOF/MS, ischemic stroke, Shengui Sansheng San

## Abstract

Cerebral energy deficiency is a key pathophysiologic cascade that results in neuronal injury and necrosis after ischemic stroke. Shengui Sansheng San (SSS) has been used to treat stroke for more than 300 years. In present study, we investigated the therapeutic efficacy and mechanism of SSS extraction on cerebral energy deficiency post-stroke. In permanent middle cerebral artery occlusion (pMCAo) model of rats, it suggested that SSS extraction in dose-dependent manner improved neurological function, cerebral blood flow (CBF), ^18^F-2-deoxy-glucose uptake and the density and diameter of alpha smooth muscle actin (α-SMA) positive vasculature in ipsilateral area, as well as decreased infarcted volume. Meanwhile, the metabolomics study in cerebrospinal fluid (CSF) was performed by using 5-(diisopropylamino)amylamine (DIAAA) derivatization-UHPLC-Q-TOF/MS approach. Eighty-eight endogenous metabolites were identified, and mainly involved in citrate cycle, fatty acid biosynthesis, aminoacyl-tRNA biosynthesis, amino acids metabolism and biosynthesis, etc. The remarkable increase of citrate in CSF after treatment with three dosages indicated that the therapeutic mechanism of SSS extraction might be related with citrate cycle. Simultaneously, it showed that high dosage group significantly increased peripheral blood glucose level, the expressions of glucose transporter (GLUT) 1, GLUT3, and monocarboxylic acid transporter 1 (MCT1), which contributed to the transportation of glucose and lactate. By the regulations of phosphorylated pyruvate dehydrogenase E1-alpha (p-PDHA1), acetyl CoA synthetase and citrate synthetase (CS), the levels of citrate and its upstream molecules (pyruvate and acetyl CoA) in peri-infarction zone further enhanced, which ultimately caused the massive yield of adenosine triphosphate (ATP). Our study first demonstrated that SSS extraction could ameliorate cerebral energy deficiency after ischemia by citrate cycle, which is characterized by the enhancements of glucose supply, transportation, utilization, and metabolism.

## Introduction

The data of the Global Burden of Diseases, Injuries, and Risk Factors Study 2010 (GBD 2010 Country Collabration, 2013) indicates that the amount of people with first stroke and stroke related deaths significantly increase from 1990 to 2010 in low-income and middle-income countries (Feigin et al., 2014). As a major type of stroke, ischemic stroke accounts for 87% (Lloyd-Jones et al., 2010). Due to the obstruction of CBF, a cascade of complex pathophysiological process emerges including energy failure, intracellular calcium overload, imbalance of ion homeostasis, neuronal excitotoxicity, inflammatory cell infiltration and so on, which ultimately leads to the neuronal apoptosis, necrosis, and neurological functional deficiency. In these pathological events, energy failure is one of the main causes of cerebral ischemic damage, because it disrupts the homeostatic mechanisms of governing the cellular volume and astrocyte/endothelial ion transport, sequentially inflicts cerebral edema and cellular death (Khanna et al., 2014; Song and Yu, 2014). Therefore, the amelioration of energy deficiency is extremely vital for neurological function after cerebral ischemia.

As the common oxidative pathway for carbohydrates, fats and amino acids, citrate cycle is the most essential process of energy metabolism in mitochondria (Akram, 2014), which generates more than 90% of the cellular energy (Chance et al., 1979; Pieczenik and Neustadt, 2007). As an energy substrate, citrate is mainly derived from glucose metabolism that is the primary form of cerebral energy (Kety, 1963). Under physiological conditions, glucose is usually catabolized into acetyl-CoA to participate in citrate cycle. Therefore, glucose metabolism is considered as the assurance for neuronal and non-neuronal cellular survival, as well as the neurotransmitters biosynthesis (Mergenthaler et al., 2013). Additionally, glucose is also used to synthesize glycogen in astrocytes, and it can be converted to lactate and transport to the neurons to protect neurological function against hypoglycemia and maintain learning processing (Hertz and Gibbs, 2009; Falkowska et al., 2015). Furthermore, the GLUTs, including GLUT1 and GLUT3, play an essential role in neuronal homeostasis (Lund-Andersen, 1979; Pardridge, 1983; McEwen and Reagan, 2004). When ischemic stroke occurs, decreased glucose delivery directly produces the severe consequences of citrate attenuation and mitochondria functional deficiency, which causes the yield of ATP to fall to less than 35% in infarcted core, and consequently advances neuronal death and irreversible cerebral injury (Hoyer and Krier, 1986; McCall, 2004; Sims and Muyderman, 2010). For these reasons, it is very crucial to promote glucose metabolism, especially citrate synthesis post-stroke.

Shengui Sansheng San is composed of *Panax ginseng* C.A.Mey., root and rhizome, *Angelica sinensis* (Oliv.) Diels, root and rhizome and *Cinnamomum cassia* (L.) J.Presl, stem bark and as an effective Chinese medical formulae, it is used to treat stroke more than 300 years. Previous studies indicate that 20(S)-ginsenoside Rg3 and ginsenoside Re played significant neuroprotective effects through enhancing mitochondrial energy metabolism and ATP generation post-stroke (Tian et al., 2005; Chen et al., 2008). *A. sinensis* exerts the actions of improving blood glucose level and glycogen reservation against physical fatigue in liver and muscle and facilitates hematopoietic function via the promotion of energy metabolism in blood deficiency mice model (Yeh et al., 2014; Hua et al., 2017). Cinnamon and its water-soluble polyphenol polymers present the feature of highly sensitive insulin-dependence in glucose metabolism, as well, cinnamon extraction enhances brain activity in obesity mouse model (Anderson et al., 2004; Gruenwald et al., 2010; Sartorius et al., 2014). Based on the observations, we hypothesized that SSS might contribute to cerebral energy metabolism after cerebral ischemia.

Metabolomics, a developing field of omics technologies, can investigate the relevant metabolites for early diagnosis and therapeutic monitoring biological systems and discover potential biomarkers of disease. Metabolites have the characteristics of structural diversity, structure similarity, and wide range of concentration. Therefore, precision analytical techniques with high-coverage, high sensitivity, and separation efficiency were required for metabolomics analysis (Want et al., 2010). As above mentioned, carbohydrates, fats, amino acids, and citrate cycle intermediates play vital roles in energy metabolism. However, the high hydrophilicity of amino acids, citrate cycle intermediates and the low molecular weights of some of them result in their hard detection, strong ion suppression and poor separation efficiency using common LC-MS method. In addition, the high hydrophobicity of long-chain fatty acids (>C6) and the trace amount of some of these fatty acids result in the poor separation and ionization efficiency (Bian et al., 2018). In our present study, DIAAA derivatization-UHPLC-Q-TOF/MS was employed to solve these difficulties and sensitively detect endogenous metabolites in CSF, which could sensitively reflect the changes of cerebral metabolism compared with serum in rats of permanent MCAo in rats. Finally, eighty-eight metabolites were determined and associated with citrate cycle, fatty acid biosynthesis, aminoacyl-tRNA biosynthesis, amino acids metabolism, and biosynthesis. Interestingly, the amounts of metabolites in citrate cycle, citrate, isocitrate, malate, and succinate, especially citrate, were found to have significant difference among MCAo model group and high dosage SSS treated group. Thereby, we focused on the cerebral glucose metabolism via citrate cycle and investigated the therapeutic efficacy and mechanism of SSS extraction on the amelioration of energy metabolism after ischemic stroke.

## Materials and Methods

### Chemical Reagents

The metabolites standards, malate, isocitrate, citrate, succinate, fumarate, α-ketoglutarate, lactate, pyruvate, isovalerate, valerate, isobutyrate, butyrate, propionate, acetate, formate, 3-hydroxybutyrate, proline, valine, methionine, histidine, isoleucine, leucine, lysine, arginine, phenylalanine, tyrosine, tryptophan, glycine, alanine, serine, aspartate, threonine, glutamate, ornithine, citrulline, anthranilic acid, 3-indolepropionic acid, 3-aminoisobutyric acid, 2-oxoproline, glycerate, lithocholic acid, isodeoxycholic acid, deoxycholic acid, cholic acid, glycoursodeoxycholic acid, palmitic acid, heptadecanoic acid, linolenic acid, linoleic acid, oleic acid, stearic acid, and dihomo-γ-linolenic acid, were purchased from Sigma-Aldrich Laboratories, Inc.

5-(Diisopropylamino)amylamine, 4-chloro-DL-phenylalanine, HATU, HOBt, TEA, and dimethyl sulfoxide (DMSO) were bought from Sigma-Aldrich Laboratories, Inc., LC-MS grade acetonitrile and HPLC grade methanol were obtained from Anaqua Chemicals Supply Inc., Ltd., (Houston, TX, United States). Deionized water was prepared using a Millipore water purification system. DIAAA was purified by prepared HPLC before use.

### The Extraction of SSS and Quality Control

Shengui Sansheng San is composed of three herbal medicines conformed to the standards of Chinese Pharmacopeia (2015), and provided by Guangzhou Zisun Pharmaceutical Co., Ltd., China. The detailed extraction method has been reported in our previously reported literature (Liu et al., 2018). Briefly, 8 kg *P. ginseng* C.A.Mey., root and rhizome, 8 kg *A. sinensis* (Oliv.) Diels, root and rhizome and 8 kg *C. cassia* (L.) J.Presl, stem bark were extracted using steam distillation, then, volatile oil and water extract were collected and mixed. Finally, 6.25 kg mixture was yielded and stored in the vacuum container at -20°C. An Agilent 1290 Infinity LC system (UHPLC, Santa Clara, CA, United States) consisting of an autosampler, DAD detector, thermostatted column compartment, and binary pump with an Agilent Eclipse XDB-C18 column (2.1 mm × 100 mm, 1.8 μm) was employed to analyze SSS extraction. The column temperature was maintained at 40°C and the autosampler was set at 4°C. The injection volume was 5 μL and the flow rate was 0.3 mL/min. Mobile phase A and B were 0.1% formic acid-containing water and 0.1% formic acid-containing acetonitrile, respectively, The gradient was set as follows: 0–1 min, 2% B; 1–6 min, 2–30% B; 6–12 min, 30–50% B; 12–17 min, 50–85% B; 17–18 min, 85–95% B; 18–21.9 min, 95% B; 22 min, 2% B.

### Middle Cerebral Artery Occlusion Model and Drug Administration

All animal operations were accorded to Guide for the Care and Use of Laboratory Animals (8th edition, Washington, DC: The National Academies Press). The experimental protocol was approved by Macau University of Science and Technology ethical committee and every experimental step of the study was performed in accordance with the regulations of animals used in research projects issued by Instituto para os Assuntos Cívicos e Municipais of Macao Special Administrative Region. Male Sprague-Dawley rats (weighing 220–250 g) supplied by the Chinese University of Hong Kong Laboratory Animal Services Center were anesthetized by 2% pentobarbital sodium (4 mL/kg, intraperitoneal injection). Subsequently, the internal and external carotid artery were dissociated, and the internal carotid artery was permanently obstructed by inserting 4-0 surgical nylon suture. During the operation, rats were placed on a heating pad to maintain their rectal temperature at 37°C. For sham-operated group, rats underwent the same surgical procedure, but without occlusion of the internal carotid artery. The rats subjected to surgery were given free access to food and water under comfortable environment (a 12: 12-h light-dark cycle).

Rats were randomly divided into five groups: sham group, MCAo group, low, middle, and high dosages of SSS groups, they were respectively, administrated with three dosages of SSS extraction (2.3 g/kg, 4.6 g/kg, and 9.2 g/kg) by gavage once daily for 3 days before surgical operation and following postoperative 7 days.

### Evaluation of Neurological Function

Neurological function was assessed by mNSS (Sughrue et al., 2006) at day 7 after ischemic stroke (*n* = 12). The detailed contents of mNSS are showed in Supplementary Table S1.

### DIAAA Derivatization-UHPLC-Q-TOF/MS Analysis in Cerebrospinal Fluid

Rats (*n* = 3) were deeply anesthetized with pentobarbital, and then 1 mL injector was carefully inserted into the posterior atlantooccipital membrane avoiding arteria spinalis dorsalis to collect 50 μL CSF from each rat. The metabolites were analyzed using our previous developed DIAAA derivatization-UHPLC-Q-TOF/MS approach (Bian et al., 2018). Briefly, 50 μL CSF were firstly mixed with 4 times volume of cold methanol to remove the proteins by centrifugation at 15,890 *g* for 5 min at 4°C. The extraction was repeated 2 times and the combined supernatants were dried under a nitrogen stream. Then, the residue was derivatized by sequentially mixing with 5 μL of 20 mM HOBt in DMSO, 5 μL of 100 mM DIAAA in DMSO containing 200 mM TEA, and 5 μL of 200 mM HATU in DMSO, and incubating for 1 min at room temperature. Finally, 35 μL acetonitrile was added to make up to the final volume of 50 and 1 μL was directly injected into UHPLC-Q-TOF/MS.

### Measurements of Infarcted Lesion Volume and Cerebral Blood Flow

A Bruker PharmaScan 7 Tesla MR scanner (Bruker BioSpin MRI GmbH, Ettlingen, Germany) using a birdcage head-coil of 75 mm inner diameter for radio frequency transmission and a 20 mm diameter surface coil for reception was used for MRI scanning. The rats (*n* = 3) at day 7 after stroke induction were anesthetized with initial inhalation of 4% isoflurane for 3 min and maintained with 2% isoflurane in a mixture of 20% oxygen and 80% room air. Prior to imaging, rats under anesthesia were placed in the stereotaxic holder of MRI machine equipped with a heating system to maintain body temperature and a pressure detector to monitor respiration. A turbo rapid acquisition with refocused echoes T_2_-weighted (T_2_WI) scan was used from Bruker BioSpin MRI GmbH: TR = 3000 ms, effective TE = 40 ms, rare factor = 4, number of average = 4; FOV = 20 mm^2^ × 20 mm^2^, slice thickness = 1 mm, matrix = 256 × 256, scan time = 6.24 min. A multi-shot DTI EPI MRI sequence was acquired with 16 contiguous axial slices thickness = 1 mm. Images were acquired with six different diffusion-encoding gradient directions with *b*-value = 1630 s/mm^2^ in the rostrocaudal direction. The in-plane resolution was 208 μm × 208 μm at a FOV of 20 mm × 20 mm and an acquisition matrix of 96 × 96, scan time = 8.24 min. Data were recorded in the same coronal and horizontal slices as chosen for T_2_WI. DSC-MRI data was acquired with GE EPI with TE = 20 ms, TR = 1000 ms, bandwidth = 250 kHz, and no averaging. 16 contiguous with 1 mm thick slices were recorded in axial orientation with a field-of-view = 20 mm × 20 mm, and a matrix of 96 × 96 to give a nominal resolution of 208 μm × 208 μm. A series of 300 images with a temporal resolution of 1000 ms were required.

### Glucose Utilization by MicroPET/CT Imaging

Positron emission tomography with ^18^F-FDG integrated with computed tomography (^18^F-FDG-PET/CT) imaging was performed to evaluate glucose uptake in ipsilateral hemisphere at day 7. After anesthetized with isoflurane (induction 3.0% in air), rats (*n* = 3) were intravenously administered with 37 MBq (∼1 mCi) of ^18^F-FDG. 60 min later, microPET/CT images were acquired for 30 min using a FLEX X-PET and X-O small animal imaging system (TriFoil). CT images were acquired with 256 projections over 2 min for attenuation correction and anatomy landmarks. PET and CT images were co-registered using commercial software (Visage Imaging) with 72 μm isotropic CT spatial resolution and 2 mm for PET imaging. For quantitative analysis, Regions of interests were drawn on the total infarcted region and boundary zone. The standardized uptake value based on body weight (SUVbw, g/ml) of four groups (MCAo, Low, Middle, and High) was detected and analyzed.

### Detection of Glucose in Peripheral Blood

The levels of glucose in peripheral blood pre-operation and post-operation 7 days were measured by Contour TS (Bayer HealthCare LLC, Mishawaka, IN, United States). Rats (*n* = 15) were anesthetized with 2% pentobarbital sodium and laied on the experimental table. 0.6 μL of blood was collected by piercing the hind toe of rats, and we put the blood drop to the edge of the test strip and recorded the data on Contour TS.

### Western Blot

At day 7, rats in five groups were sacrificed by excessive anesthesia, and transcardially perfused with 4°C saline. Brain tissues were taken from cranial cavity and protein samples (*n* = 3) were extracted. Enhanced bicinchoninic acid (BCA) protein assay kit (Beyotime Institute of Biotechnology, Shanghai, China) was used to determine protein concentration. The proteins that mixed with loading buffer were separated by 10% SDS-polyacrylamine gel and transferred to polyvinylidene fluoride membranes. Blots were probed with specific antibodies directed against GLUT1 (1:5000, Abcam), GLUT3 (1:8000, Abcam), MCT1 (1 μg/mL, Abcam), PDH (1:1000, Abcam), ACS (1:3000, Abcam), CS (1:2000, Abcam), and Beta actin (1 μg/mL, Abcam) at 4°C overnight. Subsequently, the membranes were gently washed and incubated with the fluorescent secondary antibodies at room temperature for 1 h. The signals of reactive bands were visualized by fluorescence scanner (LI-COR, Lincoln, NE, United States), and the expressions of targeted proteins in five groups were normalized against that of beta actin.

### Immunofluorescence Staining

Rats were sacrificed at day 7 and their brain tissues (*n* = 3) were fixed in 4% paraformaldehyde. Fresh frozen coronal sections of 8 μm thickness were cut by a cryostat microtome (Shandon Cryotome FSE; Thermo Fisher Scientific), and every third slice of a total sections was selected for immunofluorescence staining. Samples were incubated with primary antibody against α-SMA (5 μg/mL, Abcam) overnight at 4°C. Subsequently, Alexa Fluor 594 that anti-mouse (1:200, Molecular Probes, Life Technologies) secondary antibody was used to incubate with samples for 2 h. Nuclei were stained with Hoechst 33342 (1:5000, Molecular Probes or Jackson Laboratories) for 10 min. Fluorescent labeling was examined with a confocal laser scanning microscope (Leica TCS SP8, Zeiss, Germany). Six non-overlapping fields of one slice in the penumbral cortex were evaluated for each rat, and Image-Pro Plus 6.0 was applied to analyze the proteins’ positive signal.

### ELISA of Pyruvate, Acetyl-CoA, Citrate, and ATP

The amount of pyruvate, acetyl-CoA, citrate, and ATP were detected according to the manufacturer’s instructions of respective assay kit (*n* = 3). The absorbance values were measured at 570 nm with the SpectraMax Paradigm Multi-Mode Microplate Reader (San Jose, CA, United States) and repeated at least three times.

### Statistical Analysis

Raw data of DIAAA derivatization-UHPLC-Q-TOF/MS were extracted by MassHunter qualitative analysis software (Agilent Technologies) to find meaningful data mining. The Molecular Feature Extractor was used to extract molecular features that had identical elution profiles, and relative *m/z*-values by retention times, accurate masses, and ion intensities. Then, the relevant abundance of each metabolite was plotted by comparing with internal standard. As a consequence, using the identified metabolites as variables, PLS-DA were performed to visualize the relationship between covariance and correlation, and further obtain variables that have significant contributions to discriminate among Sham, MCAo and high dosage groups using SIMCA-P software (version 13.0; Umetrics, Umeå, Sweden). Permutation test on the responses and procedures of seven-fold cross-validation was run for each model to check its validity. Metabolic pathways were performed in the MetaboAnalyst 3.0. GraphPad Prism 5.0 (GraphPad Software, Inc., United States) was used to analyze the related data. The continuity variable results were analyzed by *t*-test, one-way ANOVA test and Tukey’s *post hoc*-test. All values were expressed as mean ± SD. Values of *p* < 0.05 was considered as statistical difference.

## Results

### Quality Control of SSS Extraction

**FIGURE 1:**
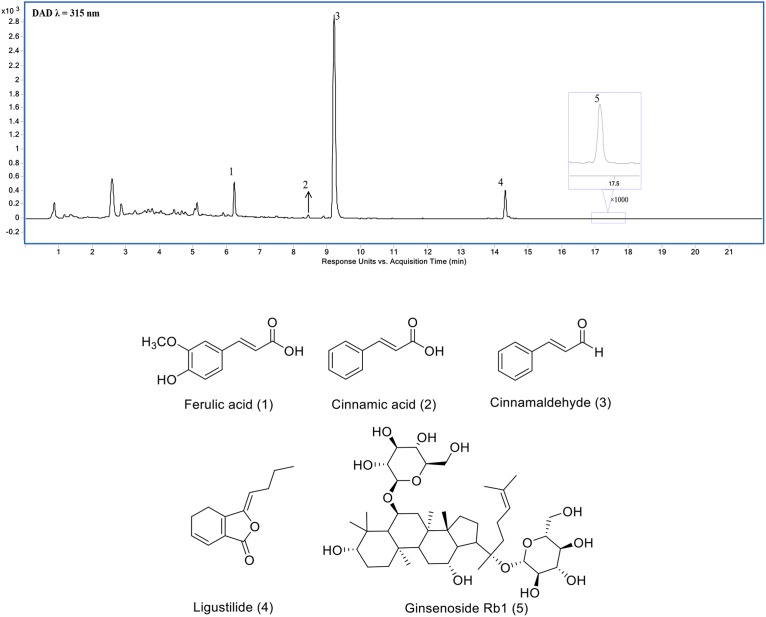
Ultra-high performance liquid chromatography (UHPLC) chromatogram of SSS extraction (1) ferulic acid, (2) cinnamic acid, (3) cinnamaldehyde, (4) Ligustilide, and (5) ginsenoside Rb1.

Three Chinese herbal medicines, *P. ginseng* C.A.Mey., root and rhizome, *A. sinensis* (Oliv.) Diels, root and rhizome, and *C. cassia* (L.) J.Presl, stem bark collectively constitute the formulae of SSS. The corresponding representative components, ginsenoside Rb1 (20.0 ± 0.4 mg/g), ligustilide (5 ± 0.3 mg/g), ferulic acid (11.7 ± 0.3 mg/g), cinnamic acid (0.1 ± 0.05 mg/g), and cinnamaldehyde (6.5 ± 0.4 mg/g) (Liu et al., 2018), were detected from SSS extraction using UHPLC method by comparison with the standards (Figure 1).

### SSS Extraction Ameliorates Neurological Function and Decreases Infarcted Volume

**FIGURE 2:**
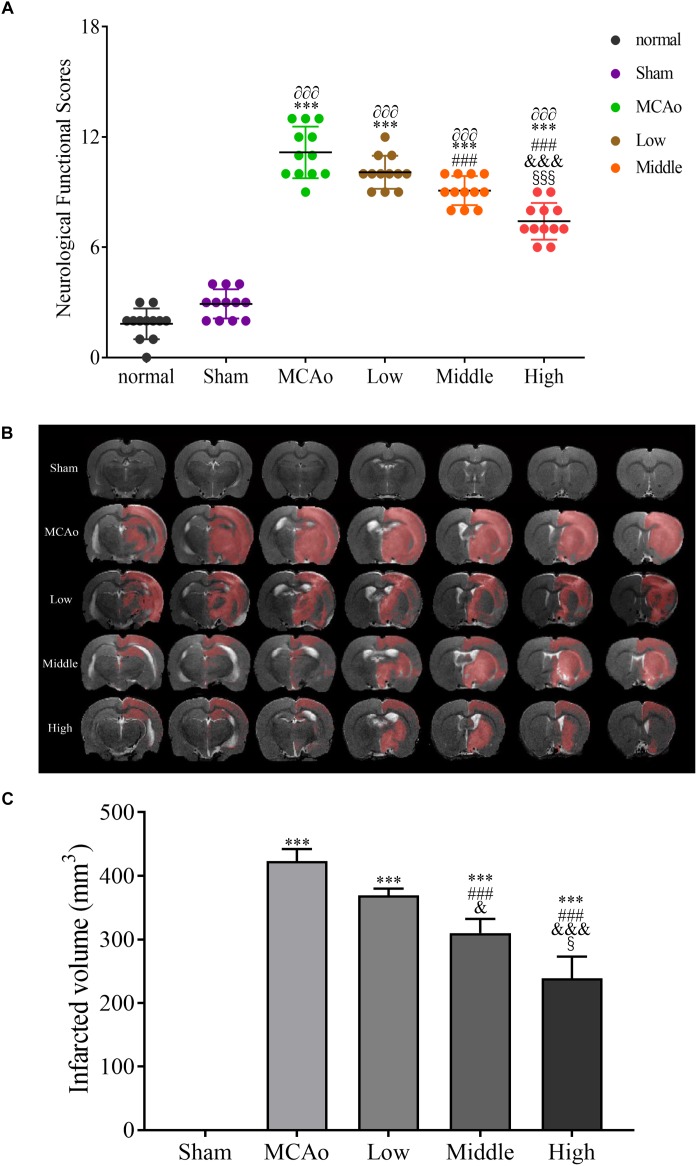
The evaluations of neurological functional scores *n* = 12 **(A)** and infarcted volume after post-surgery *n* = 3 **(B**,**C)**. Data presented as mean ± standard deviation. ^∂∂∂^*p* < 0.001 vs. normal group; ^∗∗∗^*p* < 0.001 vs. sham group; ^###^*p* < 0.001 vs. MCAo group; ^&^*p* < 0.05, ^&&&^*p* < 0.001 vs. low group; ^x^*p* < 0.05, ^xxx^*p* < 0.001 vs. middle group.

To explore the therapeutic effect of SSS on cerebral ischemia, mNSS was used to evaluate amelioration of neurological function after treatments of three dosage groups of SSS. In present study, it indicated that the high dosage group could obviously improve the neurological function compared with the low and middle dosage groups (*F* = 187.8, *P* < 0.001, Figure 2A). Additionally, infarcted volume as a severe consequence of cerebral ischemia also obviously decreased by SSS treatment whose efficacy showed a dose-dependent relationship (*F* = 190, *P* < 0.001, Figure 2B,C). These results suggested that SSS extraction could attenuate cerebral ischemic injury.

### Identification of Metabolites in CSF by DIAAA Derivatization-UHPLC-Q-TOF/MS

To determine the changes of endogenous metabolites induced by SSS treatment, the rat CSF were collected and analyzed by DIAAA derivatization-UHPLC-Q-TOF/MS.

Using the standards, the MS/MS characterization of DIAAA derivatives were firstly investigated. It was found that the characteristic MS/MS fragmentation ions of these DIAAA derivatives were [M+H-42]^+^, [M+H-84],^+^ and/or [M+H-101]^+^ derived from the neutral loss of one or two propene and/or diisopropylamino, as well, *m/z* 128 and 86 were also the characteristic ions. These fragmentation ions were related with the derivatization reagent DIAAA. Furthermore, based on the molecular formula and other fragmentation ions, the structure of unknown metabolites could be identified. For example, the fragmentation ions of citric acid at *m/z* 260.1161 and 242.1029 indicated the presence of hydroxyl. The fragment at *m/z* 214.1081 and 170.0814 demonstrated the existence of carboxyl group, which further confirmed the structure (Supplementary Figure S1A). The fragmentation ions at *m/z* 211.2070 manifested the core structure of myristic acid, in addition, the ion pairs at *m/z* 126.1281/112.1158, 112.1258/98.1043 indicated the presence of methylene groups (Supplementary Figure S1B). The fragment ions of indole-3-acetic acid at *m/z* 158.0594/130.0656 indicated the presence of carbonyl group, which also confirmed the core structure of indole-3-acetic acid (Supplementary Figure S1C).

Based on the above MS/MS information and/or comparison with the database HMDB and METLIN, as well as standards, 88 metabolites were identified from CSF. The metabolites containing the retention time, theoretical mass and measured mass were summarized in Supplementary Table S2.

### Identification of Potential Pathway of the Metabolites

**FIGURE 3:**
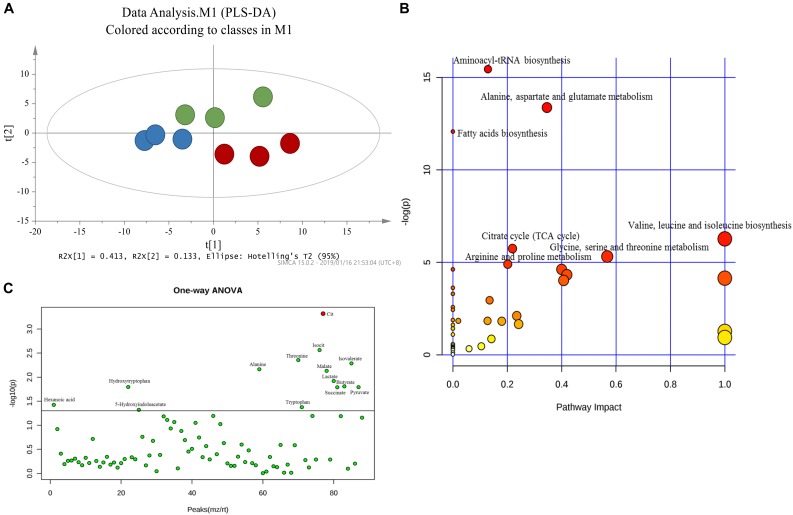
Score plot of PLS-DA **(A)** model separating high dosage group, MCAo group and sham group (Green, Sham group; Blue, MCAo group; Red, high dosage group). **(B)** Metabolic pathways involved in potential biomarkers in CSF. **(C)** One-way ANOVA analysis of identified 88 metabolites.

**FIGURE 4:**
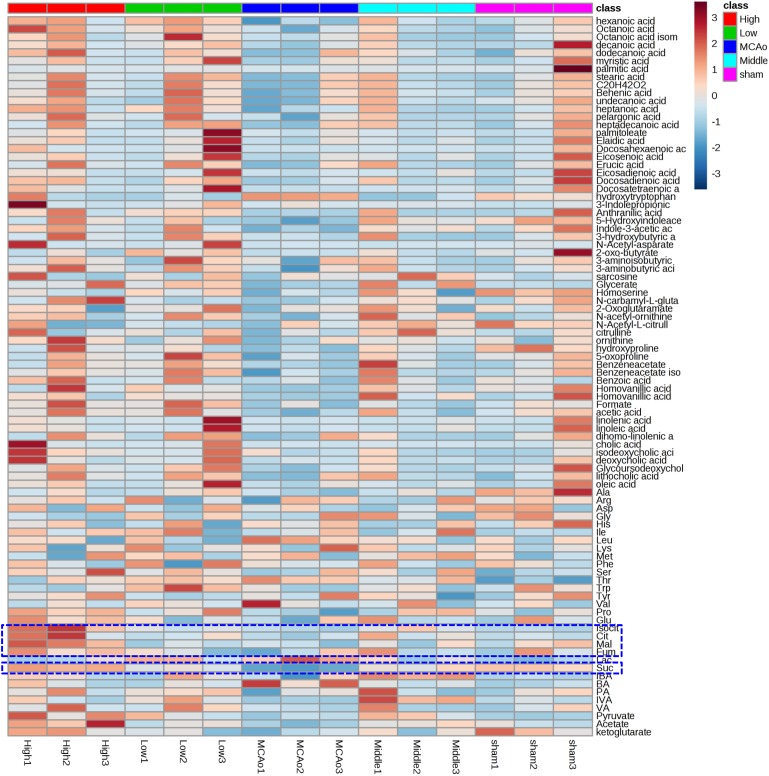
Heat maps showed 88 metabolites profiling results between MCAo and high dosage groups.

**FIGURE 5:**
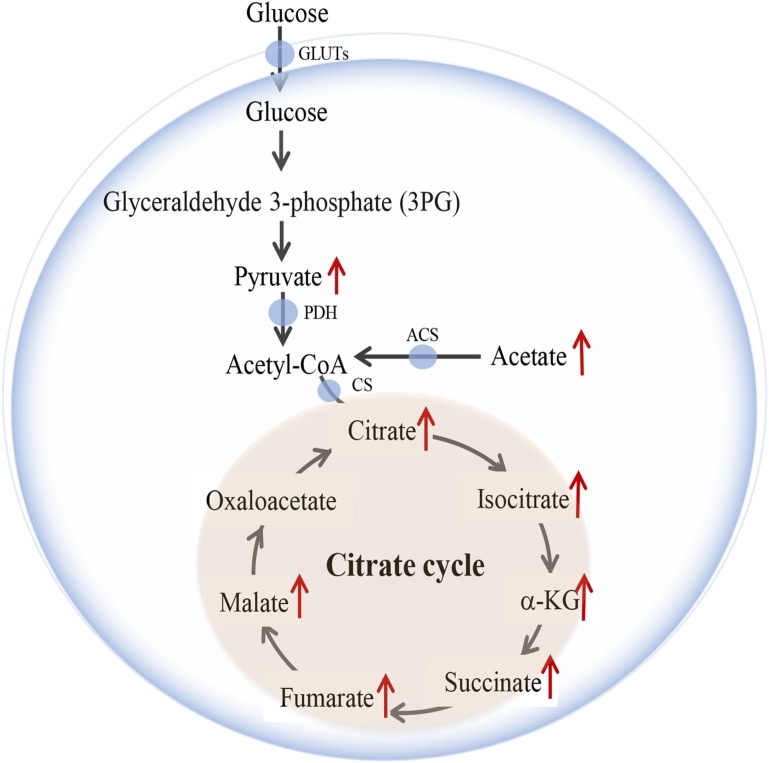
Changes of representative metabolites in metabolic pathway.

Principal component analysis was applied to capture the most significant difference among Sham, MCAo, and high dosage groups. As shown in Figure 3A, samples within groups were segregated into tight cluster, which indicated that Sham, MCAo and high dosage groups could be clearly separated with each other using PLS-DA model. Then, the metabolic pathway was established by importing these metabolites into the web-based database MetaboAnalyst. Finally, citrate cycle, fatty acids biosynthesis, aminoacyl-tRNA biosynthesis, amino acids metabolism, and biosynthesis were filtered out as the most important metabolic pathways (Figure 3B). These metabolites were then analyzed using one-way ANOVA, 14 metabolites were found to have significant differences between MCAo and high dosage groups (Figure 3C). As well, Heat map and GraphPad prism were further applied to evaluate the differential metabolites. Finally, citrate, isocitrate, malate, and succinate, especially citrate, were found to have significant difference between MCAo and high dosage groups (Figure 4, *p* < 0.01, Fold change >1.5, Supplementary Table S2). The changes of representative metabolites in the metabolic pathway were shown in Figure 5.

### SSS Extraction Increases Cerebral Blood Flow and Arteriogenesis

**FIGURE 6:**
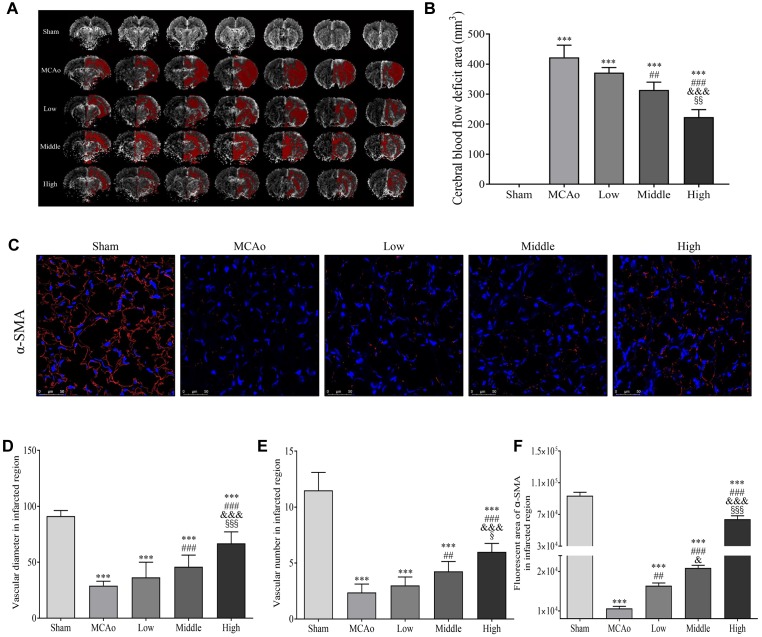
Shengui Sansheng San (SSS) extraction ameliorated the CBF deficient area and facilitated arteriogenesis (*n* = 3). **(A**,**B)** MRI imaging at day 7 and quantitative analysis of CBF deficient area. **(C)** The immunofluorescent staining of α-SMA (red) in ischemic boundary zone were presented (Magnification, ×400), and quantitative analysis of α-SMA expression, vascular density and diameter **(D–F)**. Data presented as mean ± standard deviation. ^∗∗∗^*p* < 0.001 vs. sham group; ^##^*p* < 0.01, ^###^*p* < 0.001 vs. MCAo group; ^&^*p* < 0.05, ^&&^*p* < 0.01, ^&&&^*p* < 0.001 vs. low group; ^x^*p* < 0.05, ^xx^*p* < 0.01, ^xxx^*p* < 0.001 vs. middle group.

As we know, glucose is a vital fuel of energy metabolism. Promoted cerebral blood reperfusion and abundant vascular density are required for glucose delivery in brain after stroke. As shown in Figure 6A,B, high dosage group of SSS significantly reduced CBF deficient areas in ipsilateral hemisphere compared with low and middle dosage groups and MCAo group (*p* < 0.01). Simultaneously, α-SMA expression, positive vascular density and diameter of high dosage group in ischemic boundary zone were the highest (*p* < 0.001 vs. MCAo, low and middle dosage groups, Figure 6C–F), which contributed to the promotion of CBF and glucose delivery to ischemic zone.

### SSS Extraction Improves the Concentration, Transportation, and Utilization of Glucose After Stroke

**FIGURE 7:**
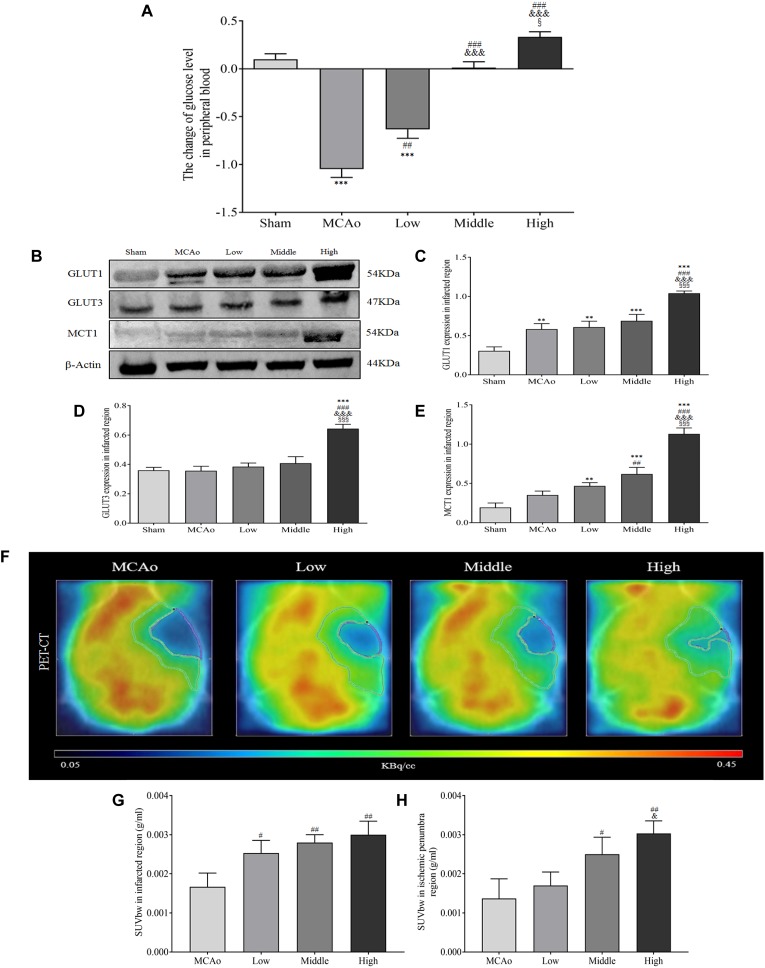
Shengui Sansheng San extraction increased the blood glucose concentration, glucose transportation and uptake by PET/CT detection after ischemic stroke. **(A)** The change of blood glucose level after 7 days by Contour TS (*n* = 15). **(B–E)** The expressions of GLUT1, GLUT3, and MCT1 in ischemic region (*n* = 3). **(F–H)** The ^18^F-FDG images and standardized uptake values were presented (*n* = 3). Data presented as mean ± standard deviation. ^∗∗∗^*p* < 0.001 vs. sham group; ^##^*p* < 0.01, ^###^*p* < 0.001 vs. MCAo group; ^&^*p* < 0.05, ^&&&^*p* < 0.001 vs. low group; ^x^*p* < 0.05, ^xxx^*p* < 0.001 vs. middle group.

The steady glucose metabolism depends on the continuous glucose concentration, transportation and uptake, which are essential for cellular survival in brain (Mergenthaler et al., 2013). Our results found that SSS extraction in dose-dependent manner enhanced glucose level of peripheral blood compared with MCAo group (*F* = 61.79, *P* < 0.001, Figure 7A). Furthermore, as shown in Figure 7B–E, high dosage group of SSS notably increased the expression of GLUT1, GLUT3, and MCT1 compared with low, middle dosages groups and MCAo group (*p* < 0.001). Due to the advantages of ameliorated CBF, increased arteriogenesis and glucose concentration, as well as the ability of glucose transportation, the characteristic glucose utilization in high dosage group was also more distinct not only in whole ischemic area but also in penumbra (*p* < 0.01 vs. MCAo, *p* < 0.05 vs. low dosage group, Figure 7F–H). Thus, these results suggested that SSS extraction provided the amount of substrate for energy metabolism by the enhancements of glucose concentration, transportation, and uptake.

### SSS Extraction Promotes ATP Yield via Citrate Cycle

**FIGURE 8:**
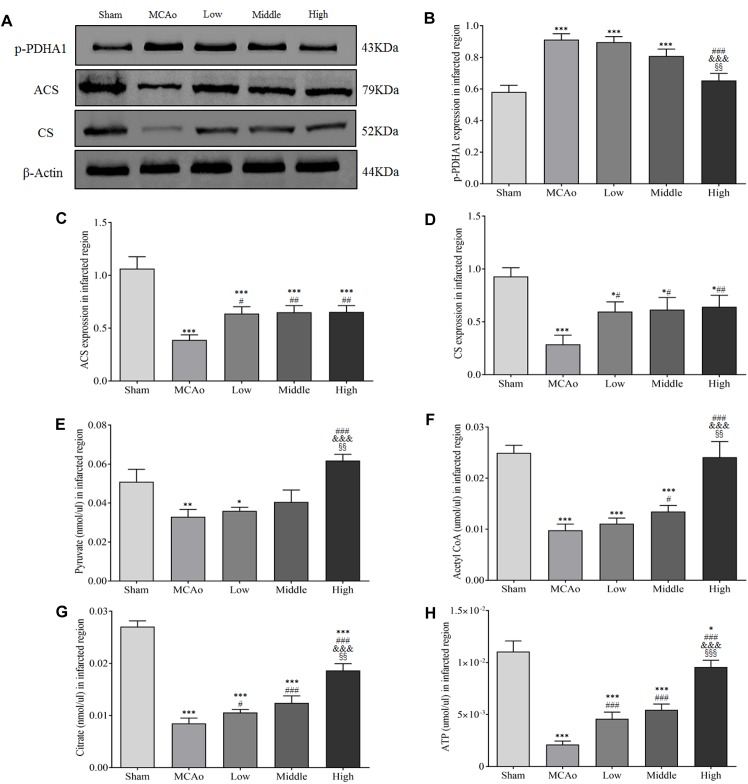
Shengui Sansheng San extraction regulated the expressions of p-PDHA1, ACS and CS and enhanced pyruvate, acetyl-CoA, citrate and ATP yields in infarcted region (*n* = 3). (**A–D**) Representative belts of western blot and quantitative analysis of p-PDHA1, ACS and CS were presented at day 7. (**E–H**) Results of pyruvate, acetyl-CoA, citrate and ATP by ELISA in infarcted region. Data presented as mean ± standard deviation. ^∗∗^*p* < 0.01, ^∗∗∗^*p* < 0.001 vs. sham group; ^#^*p* < 0.05, ^##^*p* < 0.01, ^###^*p* < 0.001 vs. MCAo group; ^&^*p* < 0.05, ^&&^*p* < 0.01 vs. low group; ^xx^*p* < 0.01, ^xxx^*p* < 0.001 vs. middle group.

Citrate cycle is crucial process of glucose metabolism, because it generates 90% cellular energy (Pieczenik and Neustadt, 2007). It demonstrates that relevant metabolism enzymes play indispensable role in citrate cycle. As shown in Figure 8A–D, western blot results indicated that SSS extraction in dose-dependent manner reduced the expression of phosphorylated pyruvate dehydrogenase E1-alpha (p-PDHA1; *F* = 38.9, *P* < 0.001). Meanwhile, the expressions of ACS and CS in three dosages groups significantly increased compared with MCAo group (*p* < 0.05). Furthermore, the results of ELISA showed that high dosage group notably enhanced some key metabolites of citrate cycle including pyruvate, acetyl-CoA, citrate and ATP in infracted region compared with low, middle dosages groups and MCAo group (*p* < 0.05, Figure 8E–H). These results suggested that SSS extraction promoted ATP production via citrate cycle, especially focused on citrate synthesis, which was parallel with the discovery by DIAAA derivatization-UHPLC-Q-TOF/MS analysis in CSF.

## Discussion

In present study, DIAAA derivatization-UHPLC-Q-TOF/MS was employed to sensitively and accurately hunt for the metabolites which could be applied to elucidate the mechanism on ischemic stroke after treatment with SSS in rats. Finally, eighty-eight metabolites were sensitively detected, they were mainly involved in citrate cycle, fatty acid biosynthesis, aminoacyl-tRNA biosynthesis, amino acids metabolism, and biosynthesis, etc. Moreover, the endogenous metabolite, citrate, which could be hardly detected using normal LC-MS method, was sensitively detected and had significant differences between MCAo and high dosage groups. This result strongly hinted that the mechanism of SSS extraction was related to citrate cycle. Subsequently, we further demonstrated that SSS extraction could enhance blood glucose level, glucose and lactate transportation, as well as glucose utilization after cerebral ischemia. Meanwhile, arteriogenesis, the improvement of CBF and relevant enzymes’ expression further contributed to citrate cycle running and ATP production. The merits of SSS extraction ameliorated neurological function deficiency and infarcted volume.

As we know, brain has high metabolic requirements consuming 20% of glucose-derived energy, while it only accounts for 2% of body weight. Citrate cycle, as the most pivotal process of energy metabolism, offers more than 90% of the energy yield (Chance et al., 1979; Pieczenik and Neustadt, 2007; Mergenthaler et al., 2013). Moreover, citrate is mainly generated from glucose metabolism and involves in citrate cycle (Kety, 1963). Under physiological conditions, glucose is usually converted to acetyl-CoA, subsequently citrate is synthesized and participates in citrate cycle for energy production. Therefore, the stabilized cerebral energy provision relies on the regular glucose metabolism. As a major energy substrate, glucose is almost fully oxidized in brain, which not only supplies ATP for neuronal and non-neuronal cellular survival, but also can be as precursor for neurotransmitters biosynthesis (Dienel, 2012; Harris et al., 2012; Howarth et al., 2012; Mergenthaler et al., 2013; Hossain et al., 2018). When cerebral ischemia occurs, the provision of glucose is insufficient, which will cause neurons necrosis in ischemic central region within a few minutes and the damage gradually extends to the surrounding zone (Dirnagl et al., 1999; Mergenthaler et al., 2004). In our study, we found that SSS extraction in dose-dependent manner increased blood glucose concentration and cerebral glucose utilization by PET/CT detection after cerebral ischemia, as well as CBF in ipsilateral hemisphere, α-SMA expression, positive vascular density and diameter in penumbra. Evidence indicated that α-SMA exerted important actions in regulations of vascular contractility and blood pressure, and the dilation of arterioles contributed to the enhancement of blood flow (Schildmeyer et al., 2000). Therefore, the improvement of cerebral glucose delivery by SSS extraction treatment is based on the increased α-SMA expression, arteriogenesis, CBF, and blood glucose concentration.

In addition to glucose delivery, cerebral glucose utilization is closely associated with correlated glucose transport proteins, including GLUT1, and GLUT3. GLUT1 is mainly expressed in endothelial cells and astrocytes, but GLUT3 is specifically existed in neurons. Though GLUT3 has a greater glucose transport ratio, neurons prefer to utilize lactate derived from astrocytes for producing energy (Simpson et al., 2007; Magistretti and Allaman, 2015). In astrocytes, lactate is usually transported to neurons by MCT1 (Magistretti and Allaman, 2015). Furthermore, study showed that neuronal loss would be induced without MCT1 (Lee et al., 2012). Meanwhile, GLUT1 and GLUT3 are also extremely essential for maintaining the neuronal homeostasis (Lund-Andersen, 1979; Pardridge, 1983; McEwen and Reagan, 2004). The expression of GLUT1 and GLUT3 could significantly remedy the energy defect after cerebral ischemia (Lee and Bondy, 1993; Espinoza-Rojo et al., 2010). Therefore, the amelioration of correlated glucose transport proteins is very vital for attenuating cerebral injury. In present study, we demonstrated that SSS extraction effectively enhanced the expression of GLUT1 and GLUT3 in ischemic boundary zone at day 7 after cerebral ischemia, which meant that more glucose was successfully transported into neuronal and non-neuronal cells. Simultaneously, the expression of MCT1 also enhanced by SSS extraction treatment, suggesting SSS extraction was beneficial to lactate shuttle and neuronal survival. These results revealed that SSS extraction offered abundant energy substrates against cerebral ischemic injury.

As the actuators of glucose oxidative metabolism, PDH and CS are exceeding key for energy production. PDH is a gatekeeper enzyme in glucose oxidative metabolism, which is consisted of PDHA1, dihydrolipoyl transacetylase, and dihydrolipoyl dehydrogenase (Ozden et al., 2014). When the oxygen and nutrient reduce, PDHA1 is phosphorylated, which will inhibit PDH activity and acetyl-CoA synthesis, and ultimately disturb the energy metabolism (Robinson, 1993; Pilegaard et al., 2006; Patel et al., 2012). CS, a catalyst, mainly promotes citrate synthesis by utilizations of acetyl-CoA and oxaloacetate. If CS silences, citrate synthesis will decrease, which induces the disorder of cellular respiratory and inefficient production of ATP (Nelson and Cox, 2008; Lin et al., 2012). Moreover, under the hypoxic condition, glucose oxidative metabolism is weakened, which causes acetyl-CoA synthesis to decline. However, acetyl-CoA can be synthesized by ACS to attenuate the deficiency of glucose oxidative metabolism. Our results indicated that SSS extraction decreased the expression of p-PDHA1 after cerebral ischemia, which contributed to the promotion of PDH activity. Additionally, the expression of ACS and CS also elevated through SSS extraction treatment. Moreover, the crucial metabolic components in citrate cycle, including pyruvate, acetyl-CoA and citrate, also notably increased. Importantly, SSS extraction administration remarkably promoted ATP yield in infracted region, especially high dosage of SSS. These results showed that SSS extraction could promote cerebral energy generation via citrate cycle, which was favorable to relieve neurological functional injury and infarcted volume. In this study, as Chinese medical formulae, SSS exerts obvious effect on the amelioration of energy metabolism after stroke. Evidence indicated that ginseng extraction could promote the utilization of glucose in rats after cerebral ischemia (Choi et al., 1996). Ginsenoside Rg1 and ginsenoside Rb1 increased GLUT3 expression and ATP yield in mice after ischemic stroke, and Rb1 transportation across blood–brain barrier (BBB) was partly regulated by GLUT1 on microvascular endothelial cells (Huang et al., 2015). In present study, high expression of GLUT1 by SSS should make Rb1 easy to pass BBB after cerebral ischemia. Ginsenoside Rd and cinnamomum polyphenols could ameliorate the mitochondrial dysfunction after cerebral ischemia, indicating that the energy metabolic defect was improved (Panickar et al., 2009; Ye et al., 2011). Additionally, *A. sinensis* polysaccharides increased the levels of blood glucose and glycogenic activity, and ligustilide and ferulic acid were apt to transport across BBB (Zhang, 2015). These ingredients are all included in SSS, so we postulated that the improvement of energy metabolism by SSS should attribute to synthesized outcome of active compounds in three herbal medicines. Of course, the pharmacokinetics of related compounds will be our further investigation in order to clarify effective ingredients of SSS.

## Conclusion

In summary, SSS extraction significantly ameliorates cerebral energy metabolism via boosting citrate cycle, which mainly embodies the enhancements of blood glucose concentration, glucose and lactate transportation and glucose utilization, as well as the regulations of relative enzymes activities in citrate cycle. These ameliorations ultimately resulted in numerous ATP yield after stroke, which improved neurological function and infarcted volume. Collectively, it suggests that SSS extraction has exerted advantageous effect in the treatment of cerebral ischemia.

## Ethics Statement

All animal operations were accorded to Guide for the Care and Use of Laboratory Animals (8th edition, Washington, DC, United States: The National Academies Press). The experimental protocol was approved by Macau University of Science and Technology ethical committee and every experimental step of the study was performed in accordance with the regulations of animals used in research projects issued by Instituto para os Assuntos Cívicos e Municipais of Macao Special Administrative Region.

## Author Contributions

YZ and J-LW designed the research. CL, ZX, and BL evaluated neurological function, performed western blot, immunofluorescence staining, and ELISA in animals experiments. XB completed quality control and performed DIAAA Derivatization-UHPLC-Q-TOF/MS test. QZ and CK finished MRI and MicroPET/CT detections and analysis. QC tested the blood glucose level. CL and XB wrote the manuscript.

## Conflict of Interest Statement

The authors declare that the research was conducted in the absence of any commercial or financial relationships that could be construed as a potential conflict of interest. The reviewer JW declared a shared affiliation, with no collaboration, with the authors to the handling Editor at the time of review.

## Abbreviations

α-SMA: alpha smooth muscle actin
ACS: acetyl-CoA synthetase
ATP: adenosine triphosphate
CBF: cerebral blood flow
CS: citrate synthetase
CSF: cerebrospinal fluid
DIAAA: 5-(diisopropylamino)amylamine
EPI: echo-planar imaging
^18^F-FDG: 2-deoxy-2-[fluorine-18] fluoro-D-glucose
FOV: field of view
GLUT: glucose transporter
HATU: *O*-(7-azabenzotriazol-1-yl)-*N*,*N*,*N*’,*N*’-tetramethyluronium hexafluorophosphate
HOBt: 1-hydroxybenzotriazole hydrate
MCAo: middle cerebral artery occlusion
MCT1: monocarboxylic acid transporter 1
mNSS: modified neurological severity score
MRI: magnetic resonance imaging
PLS-DA: Partial least squares discriminant analysis
PET/CT: positron emission tomography/computed tomography
p-PDHA1: phosphorylated pyruvate dehydrogenase E1-alpha
SSS: Shengui Sansheng San
TE: echo time
TEA: triethylamine
TR: repetition time
UHPLC-Q-TOF/MS: ultra-high performance liquid chromatography-quadrupole-time of flight mass spectrometry
