# Supplementary material for: Shengui Sansheng San Ameliorates Cerebral Energy Deficiency via Citrate Cycle After Ischemic Stroke
Source: Front Pharmacol. 2019 Apr 23;10:386. doi: 10.3389/fphar.2019.00386 (PMC6489525; doi:10.3389/fphar.2019.00386)
Supplement: Supplementary file 1 [file Data_Sheet_1.docx]

**Shengui Sansheng San Ameliorates Cerebral Energy Deficiency Via Citrate Cycle after Ischemic Stroke**

Cheng Luo^a,#^, Xiqing Bian^a,#^, Qian Zhang^b^, Zhenyan Xia^a^, Bowen Liu^a^, Qi Chen^a^, Chienchih Ke^c,d^, Jian-Lin Wu^a,*^, Yonghua Zhao^e,*^

^a^ State Key Laboratory of Quality Research in Chinese Medicine, Faculty of Chinese Medicine, Macau University of Science and Technology, Macao

^b^ Department of Biotherapy, Shenzhen Luo hu People's Hospital, Shenzhen, PR China

^c^ Department of Medical Imaging and Radiological Sciences, Kaohsiung Medical University, Kaohsiung, Taiwan

^d^ Biomedical Imaging Research Center, National Yang-Ming University, Taipei, Taiwan

^e^ State Key Laboratory of Quality Research in Chinese Medicine, Institute of Chinese Medical Sciences, University of Macau, Macao

^#^ Both authors contributed equally to this study.

*Corresponding author

Yonghua Zhao, M.D.

yonghuazhao@um.edu.mo

Jian-Lin Wu, PhD.

[jlwu@must.edu.mo](mailto:jlwu@must.edu.mo)

**Table of content:**

**Figure S1** MS/MS spectra for DIAAA derivatives of citrate (A), myristic acid (B), and indole-3-acetic acid (C).

**Table S1** Motor tests

**Table S2** List of identified metabolites in cerebrospinal fluid (CSF)


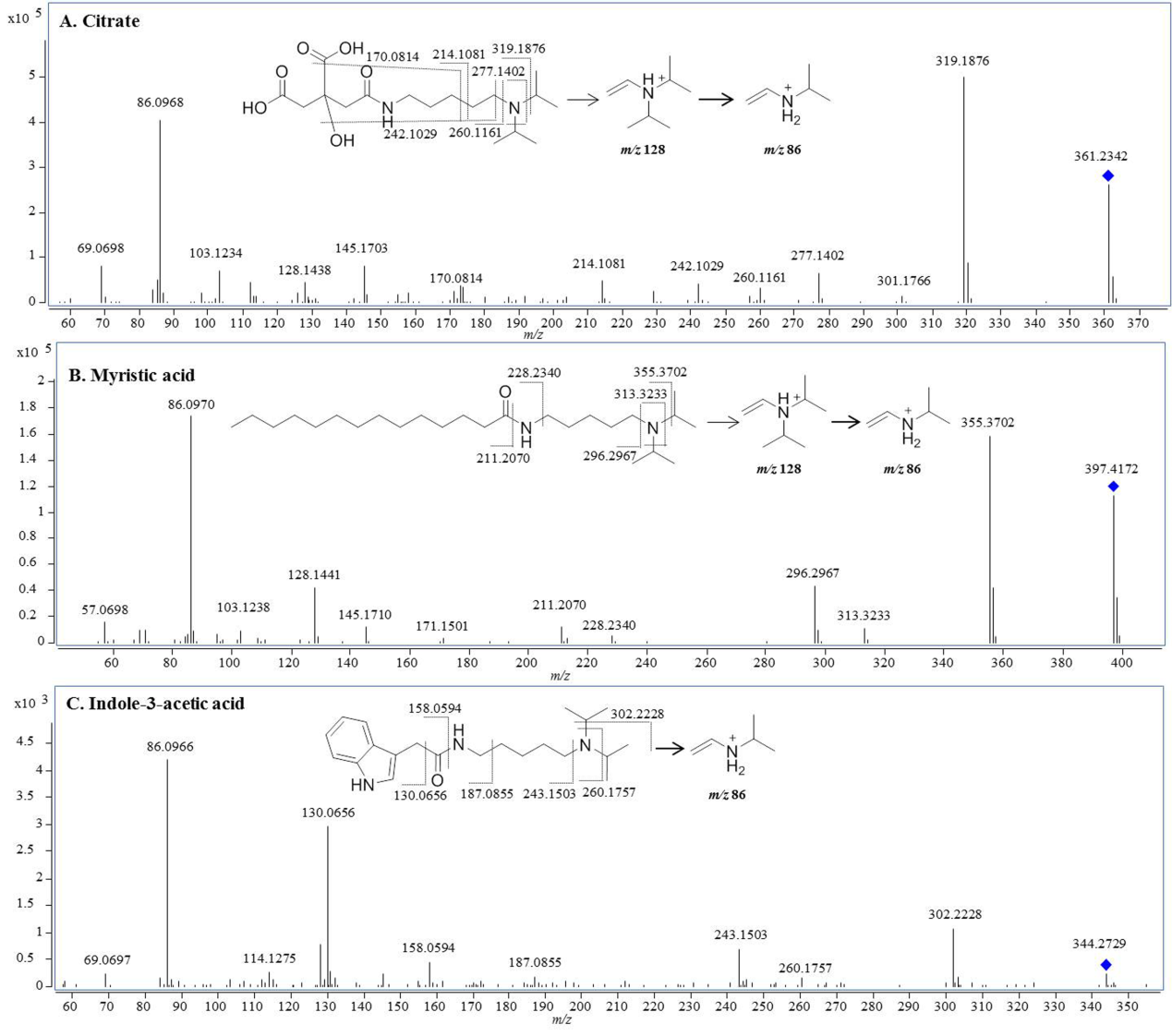


Figure S1 MS/MS spectra for DIAAA derivatives of citrate (A), myristic acid (B), and indole-3-acetic acid (C).

| **Table S1** Motor tests | |
| --- | --- |
| Raising rat by tail | 3 |
| Flexion of forelimb | 1 |
| Flexion of hindlimb | 1 |
| Head moved > 10° to vertical axis within 30s | 1 |
| Placing rat on floor (normal=0; maximum=3) | 3 |
| Normal walk | 0 |
| Inability to walk straight | 1 |
| Circling toward the paretic side | 2 |
| Fall down to the paretic side | 3 |
| Sensory tests | 2 |
| Placing test (visual and tactile test) | 1 |
| Proprioceptive test (deep sensation, pushing paw against table edge to stimulate limb muscles) | 1 |
| Beam balance tests (normal=0; maximum=6) | 6 |
| Balances with steady posture | 0 |
| Grasps side of beam | 1 |
| Hugs beam and 1 limb falls down from beam | 2 |
| Hugs beam and 2 limbs fall down from beam, or spins on beam (> 60s) | 3 |
| Attempts to balance on beam but falls off (> 40s) | 4 |
| Attempts to balance on beam but falls off (> 20s) | 5 |
| Falls off, No attempt to balance or hang on to beam (20s) | 6 |
| Reflex absence and abnormal movements | 4 |
| Pinna reflex (head shake when auditory meatus is touched) | 1 |
| Corneal reflex (eye blink when cornea is lightly touched with cotton) | 1 |
| Startle reflex (motor response to a brief noise from snapping a clipboard paper) | 1 |
| Seizures, myoclonus, myodystony | 1 |
| Maximum points | 18 |
| One point is awarded for the inability to perform the tasks or for lack of a tested reflex, 13–18, severe injury; 7–12, moderate injury; 1–6, mild injury. | |

**Table S2** List of identified metabolites in cerebrospinal fluid (CSF)

| **No.** | **Metabolites** | **Fold Change (High/MCAo)** | **P value (MCAo&High)** | **Theoretical mass ([M+H]^+^)** | **Measured mass ([M+H]^+^)** | **Error (ppm)** | **RT (min)** | **Formula** | **Derivatization formula** | **HMDB** | **KEGG** | **Pathway** |
| --- | --- | --- | --- | --- | --- | --- | --- | --- | --- | --- | --- | --- |
| 1 | Malate | 1.74 | 0.0081 | 303.2278 | 303.2284 | -3.08 | 4.54 | C_4_H_6_O_5_ | C_15_H_30_N_2_O_4_ | HMDB00744 | C00149 | Citrate cycle |
| 2 | Isocitrate | 2.78 | 0.010 | 361.2333 | 361.2341 | -2.43 | 3.86 | C_6_H_8_O_7_ | C_17_H_32_N_2_O_6_ | HMDB00193 | C00311 | Citrate cycle |
| 3 | Citrate | 5.81 | 0.0015 | 361.2333 | 361.2339 | -1.97 | 4.56 | C_6_H_8_O_7_ | C_17_H_32_N_2_O_6_ | HMDB00094 | C00158 | Citrate cycle |
| 4 | Succinate | 1.78 | 0.0013 | 287.2329 | 287.2338 | -3.80 | 5.73 | C_4_H_6_O_4_ | C_15_H_30_N_2_O_3_ | HMDB00254 | C00042 | Citrate cycle or Alanine, aspartate and glutamate metabolism |
| 5 | Fumarate | 1.57 | 0.14 | 285.2173 | 285.2177 | -1.66 | 8.09 | C_4_H_4_O_4_ | C_15_H_28_N_2_O_3_ | HMDB00134 | C00122 | Citrate cycle or Alanine, aspartate and glutamate metabolism or Arginine and proline metabolism |
| 6 | Acetate | 1.70 | 0.058 | 229.2274 | 229.228 | -2.91 | 4.77 | C_2_H_4_O_2_ | C_13_H_28_N_2_O | HMDB00042 | C00033 | Pyruvate metabolism |
| 7 | Lactate | -1.92 | 0.029 | 259.238 | 259.2384 | -1.75 | 5.01 | C_3_H_6_O_3_ | C_14_H_30_N_2_O_2_ | HMDB00190 | C00186 | Pyruvate metabolism |
| 8 | Pyruvate | 5.31 | 0.039 | 257.2224 | 257.2233 | -3.26 | 8.83 | C_3_H_4_O_3_ | C_14_H_28_N_2_O_2_ | HMDB00243 | C00022 | Pyruvate metabolism or Alanine, aspartate and glutamate metabolism or Glycine, serine and threonine metabolism |
| 9 | Proline | 1.67 | 0.0066 | 301.2598 | 301.2578 | 6.81 | 1.09 | C_5_H_9_NO_2_ | C_16_H_33_N_3_O | HMDB00162 | C00148 | Aminoacyl-tRNA biosynthesis |
| 10 | Valine | 1.02 | 0.77 | 286.2853 | 286.2862 | -2.87 | 2.28 | C_5_H_11_NO_2_ | C_16_H_35_N_3_O | HMDB00883 | C00183 | Aminoacyl-tRNA biosynthesis |
| 11 | Methionine | 1.02 | 0.88 | 318.2574 | 318.2578 | -1.46 | 4.87 | C_5_H_11_NO_2_S | C_16_H_35_N_3_OS | HMDB00696 | C00073 | Aminoacyl-tRNA biosynthesis |
| 12 | Histidine | -1.10 | 0.40 | 324.2758 | 324.2772 | -4.40 | 0.99 | C_6_H_9_N_3_O_2_ | C_17_H_33_N_5_O | HMDB00177 | C00135 | Aminoacyl-tRNA biosynthesis |
| 13 | Isoleucine | 1.14 | 0.58 | 300.3009 | 300.3016 | -1.48 | 3.06 | C_6_H_13_NO_2_ | C_17_H_37_N_3_O | HMDB00172 | C00407 | Aminoacyl-tRNA biosynthesis |
| 14 | Leucine | -1.28 | 0.11 | 300.3009 | 300.3013 | -0.54 | 3.28 | C_6_H_13_NO_2_ | C_17_H_37_N_3_O | HMDB00687 | C00123 | Aminoacyl-tRNA biosynthesis |
| 15 | Lysine | -1.08 | 0.62 | 315.3129 | 315.3124 | -1.75 | 6.53 | C_6_H_14_N_2_O_2_ | C_17_H_38_N_4_O | HMDB00182 | C00047 | Aminoacyl-tRNA biosynthesis |
| 16 | Arginine | 1.03 | 0.87 | 343.318 | 343.3181 | -1.53 | 0.99 | C_6_H_14_N_4_O_2_ | C_17_H_38_N_6_O | HMDB00517 | C00062 | Aminoacyl-tRNA biosynthesis |
| 17 | Phenylalanine | 1.09 | 0.29 | 334.2853 | 334.2867 | -3.56 | 9.96 | C_9_H_11_NO_2_ | C_20_H_35_N_3_O | HMDB00159 | C00079 | Aminoacyl-tRNA biosynthesis |
| 18 | Tyrosine | -1.06 | 0.59 | 350.2802 | 350.2803 | -0.57 | 5.11 | C_9_H_11_NO_3_ | C_20_H_35_N_3_O_2_ | HMDB00158 | C00082 | Aminoacyl-tRNA biosynthesis |
| 19 | Tryptophan | 1.01 | 0.87 | 373.2962 | 373.2962 | 0.11 | 13.58 | C_11_H_12_N_2_O_2_ | C_22_H_36_N_4_O | HMDB00929 | C00078 | Aminoacyl-tRNA biosynthesis |
| 20 | Glycine | -1.20 | 0.34 | 244.238 | 244.237 | 5.22 | 1.08 | C_2_H_5_NO_2_ | C_13_H_29_N_3_O | HMDB00123 | C00037 | Aminoacyl-tRNA biosynthesis or Glycine, serine and threonine metabolism or Methane metabolism |
| 21 | Alanine | 1.14 | 0.20 | 258.254 | 258.2555 | -5.53 | 1.53 | C_3_H_7_NO_2_ | C_14_H_31_N_3_O | HMDB00161 | C00041 | Aminoacyl-tRNA biosynthesis or Alanine, aspartate and glutamate metabolism |
| 22 | Serine | 1.58 | 0.27 | 274.2483 | 274.2468 | 4.57 | 1.07 | C_3_H_7_NO_3_ | C_14_H_31_N_3_O_2_ | HMDB00187 | C00065 | Aminoacyl-tRNA biosynthesis or Glycine, serine and threonine metabolism or Methane metabolism |
| 23 | Asparate | 1.06 | 0.65 | 301.2598 | 301.2547 | -3 | 6.23 | C_4_H_7_NO_4_ | C_15_H_31_N_3_O_3_ | HMDB00191 | C16433 | Aminoacyl-tRNA biosynthesis or Alanine, aspartate and glutamate metabolism or Arginine and proline metabolism |
| 24 | Threonine | 1.33 | 0.13 | 288.2646 | 288.2699 | -3.84 | 1.09 | C_4_H_9_NO_3_ | C_15_H_33_N_3_O_2_ | HMDB00167 | C00188 | Aminoacyl-tRNA biosynthesis or Glycine, serine and threonine metabolism |
| 25 | Aminobutyrate | 1.55 | 0.17 | 272.2696 | 272.27 | 0.25 | 13.39 | C_4_H_9_NO_2_ | C_15_H_33_N_3_O | - | - | Alanine, aspartate and glutamate metabolism |
| 26 | α-Ketoglutarate | 1.69 | 0.046 | 315.2278 | 315.2276 | -5.14 | 4.68 | C_5_H_6_O_5_ | C_16_H_30_N_2_O_4_ | HMDB00208 | C00026 | Alanine, aspartate and glutamate metabolism |
| 27 | Oxoglutaramic acid | 1.05 | 0.84 | 314.2438 | 314.2447 | -4.31 | 7.33 | C_5_H_7_NO_4_ | C_16_H_31_N_3_O_3_ | HMDB01552 | C00940 | Alanine, aspartate and glutamate metabolism |
| 28 | Glutamate | 1.57 | 0.14 | 316.2595 | 316.2536 | 5.71 | 1.25 | C_5_H_9_NO_4_ | C_16_H_33_N_3_O_3_ | HMDB00148 | C00025 | Alanine, aspartate and glutamate metabolism |
| 29 | N-acetyl-asparate | 1.34 | 0.25 | 344.254 | 344.2551 | -1 | 4.61 | C_6_H_9_NO_5_ | C_17_H_33_N_3_O_4_ | HMDB00812 | C01042 | Alanine, aspartate and glutamate metabolism |
| 30 | Anthranilic acid | 15.25 | 0.10 | 306.254 | 306.2544 | -1.23 | 9.12 | C_7_H_7_NO_2_ | C_18_H_31_N_3_O | HMDB01123 | C00108 | Tryptophan metabolism |
| 31 | Indole-3-acetic acid | 2.79 | 0.027 | 344.2696 | 344.2704 | 1.89 | 18.76 | C_10_H_9_NO_2_ | C_21_H_33_N_3_O | HMDB00197 | C00954 | Tryptophan metabolism |
| 32 | 5-Hydroxyindoleacetate | 2.19 | 0.061 | 360.2646 | 360.2656 | -2.5 | 13.41 | C_10_H_9_NO_3_ | C_21_H_33_N_3_O_2_ | HMDB00763 | C05635 | Tryptophan metabolism |
| 33 | 3-Indolepropionic acid | -1.76 | 0.51 | 358.2891 | 358.2855 | -0.08 | 20.30 | C_11_H_11_NO_2_ | C_22_H_35_N_3_O | HMDB02302 | - | Tryptophan metabolism |
| 34 | Hydroxytryptophan | -1.59 | 0.00060 | 389.2911 | 389.2945 | -7.74 | 15.88 | C_11_H_12_N_2_O_3_ | C_22_H_36_N_4_O_2_ | HMDB00472 | C01017 | Tryptophan metabolism |
| 35 | Homovanillic acid isomer | 2.24 | 0.0042 | 351.2642 | 351.2643 | -0.24 | 13.73 | C_9_H_10_O_4_ | C_20_H_34_N_2_O_3_ | - | - | Tyrosine metabolism |
| 36 | Homovanillic acid | 1.94 | 0.086 | 351.2642 | 351.2646 | -2.19 | 14.57 | C_9_H_10_O_4_ | C_20_H_34_N_2_O_3_ | HMDB00118 | C05582 | Tyrosine metabolism |
| 37 | Benzoic acid | 1.64 | 0.13 | 291.2431 | 291.2437 | -2.61 | 17.28 | C_7_H_6_O_2_ | C_18_H_30_N_2_O | HMDB01870 | C00180 | Phenylalanine metabolism |
| 38 | Benzeneacetate | 1.50 | 0.098 | 305.2587 | 305.2595 | -2.32 | 18.02 | C_8_H_8_O_2_ | C_19_H_32_N_2_O | HMDB00209 | C07086 | Phenylalanine metabolism |
| 39 | Benzeneacetate isomer | 1.46 | 0.072 | 305.2587 | 305.2598 | -3.37 | 19.96 | C_8_H_8_O_2_ | C_19_H_32_N_2_O | - | - | Phenylalanine metabolism |
| 40 | Hydroxyproline | 2.17 | 0.14 | 300.2646 | 300.2657 | -3.66 | 5.32 | C_5_H_9_NO_3_ | C_16_H_33_N_3_O_2_ | HMDB00725 | C01157 | Arginine and proline metabolism |
| 41 | Ornithine | 1.52 | 0.12 | 301.2962 | 301.2968 | -1.5 | 0.96 | C_5_H_12_N_2_O_2_ | C_16_H_36_N_4_O | HMDB00214 | C00077 | Arginine and proline metabolism |
| 42 | Citrulline | 1.41 | 0.39 | 344.302 | 344.3018 | -0.43 | 1.60 | C_6_H_13_N_3_O_3_ | C_17_H_37_N_5_O_2_ | HMDB00904 | C00327 | Arginine and proline metabolism |
| 43 | N-acetyl-ornithine | 1.66 | 0.17 | 343.3068 | 343.3073 | -1.52 | 13.78 | C_7_H_14_N_2_O_3_ | C_18_H_38_N_4_O_2_ | HMDB03357 | C00437 | Arginine and proline metabolism |
| 44 | N-acetyl-L-citrulline | 1.04 | 0.85 | 386.3126 | 386.3131 | -1.64 | 6.42 | C_8_H_15_N_3_O_4_ | C_19_H_39_N_5_O_3_ | HMDB00856 | C15532 | Arginine and proline metabolism |
| 45 | Sarcosine | -1.85 | 0.15 | 258.254 | 258.2548 | -2.42 | 2.10 | C_3_H_7_NO_2_ | C_14_H_31_N_3_O | HMDB00271 | C00213 | Glycine, serine and threonine metabolism |
| 46 | Glycerate | 2.50 | 0.097 | 275.2329 | 275.2334 | -1.96 | 3.25 | C_3_H_6_O_4_ | C_14_H_30_N_2_O_3_ | HMDB00139 | C00258 | Glycine, serine and threonine metabolism |
| 47 | Homoserine | 1.13 | 0.26 | 288.2646 | 288.265 | -1.03 | 1.62 | C_4_H_9_NO_3_ | C_15_H_33_N_3_O_2_ | HMDB00719 | C00263 | Glycine, serine and threonine metabolism |
| 48 | Oxo-butyrate | 2.01 | 0.065 | 271.238 | 271.2388 | -2.73 | 5.96 | C_4_H_6_O_3_ | C_15_H_30_N_2_O_2_ | - | - | Cysteine and methionine metabolism or Propanoate metabolism |
| 49 | Formate | 1.57 | 0.14 | 215.2118 | 215.2124 | -3.18 | 3.89 | CH_2_O_2_ | C_12_H_26_N_2_O | HMDB00142 | C00058 | Methane metabolism |
| 50 | Oxoproline | 1.35 | 0.12 | 298.2495 | 298.2498 | -3.28 | 4.87 | C_5_H_7_NO_3_ | C_16_H_32_N_3_O_2_ | - | - | Glutathione metabolism |
| 51 | N-carbamyl-L-glutamate | 2.09 | 0.077 | 331.2591 | 331.2595 | -1.32 | 7.56 | C_6_H_10_N_2_O_5_ | C_17_H_34_N_2_O_4_ | HMDB15673 | C05829 | Histidine metabolism |
| 52 | 3-Aminoisobutyric acid | 1.14 | 0.64 | 272.2696 | 272.2706 | -3.19 | 12.55 | C_4_H_9_NO_2_ | C_15_H_33_N_3_O | HMDB03911 | C05145 | Pyrimidine metabolism |
| 53 | Lithocholic acid | 1.36 | 0.27 | 545.5041 | 545.5036 | 0.74 | 37.21 | C_24_H_40_O_3_ | C_35_H_64_N_2_O_2_ | HMDB00761 | C03990 | Primary bile acid biosynthesis |
| 54 | Isodeoxycholic acid | 2.56 | 0.17 | 561.499 | 561.497 | 1.81 | 29.11 | C_24_H_40_O_4_ | C_35_H_64_N_2_O_3_ | HMDB02536 | C17661 | Primary bile acid biosynthesis |
| 55 | Deoxycholic acid | 1.09 | 0.86 | 561.499 | 561.4982 | 1.7 | 32.54 | C_24_H_40_O_4_ | C_35_H_64_N_2_O_3_ | HMDB00626 | C04483 | Primary bile acid biosynthesis |
| 56 | Cholic acid | 4.44 | 0.087 | 577.4944 | 577.4939 | 0.44 | 29.16 | C_24_H_40_O_5_ | C_35_H_64_N_2_O_4_ | HMDB00619 | C00695 | Primary bile acid biosynthesis |
| 57 | Glycoursodeoxycholic acid | 1.53 | 0.098 | 618.5204 | 618.5205 | 1.2 | 28.16 | C_26_H_43_NO_5_ | C_37_H_67_N_3_O_4_ | HMDB00708 | - | Primary bile acid biosynthesis |
| 58 | Isobutyrate | 1.25 | 0.45 | 257.2587 | 257.2599 | -4.29 | 11.25 | C_4_H_8_O_2_ | C_15_H_32_N_2_O | HMDB01873 | C02632 | Short chain fatty acids metabolism |
| 59 | Isovalerate | 1.78 | 0.16 | 271.2744 | 271.2753 | -2.93 | 15.97 | C_5_H_10_O_2_ | C_16_H_34_N_2_O | HMDB00718 | C08262 | Short chain fatty acids metabolism |
| 60 | Valerate | 1.30 | 0.22 | 271.2744 | 271.2751 | -2.51 | 16.24 | C_5_H_10_O_2_ | C_16_H_34_N_3_O | HMDB00892 | C00803 | Short chain fatty acids metabolism |
| 61 | Propionate | 1.23 | 0.33 | 243.2431 | 243.2443 | -0.09 | 7.31 | C_3_H_6_O_2_ | C_14_H_30_N_2_O | HMDB00237 | C00163 | Propanoate metabolism |
| 62 | Butyrate | -2.07 | 0.12 | 257.2587 | 257.2599 | -4.12 | 11.93 | C_4_H_8_O_2_ | C_15_H_32_N_2_O | HMDB00039 | C00246 | Butanoate metabolism |
| 63 | 3-Hydroxybutyrate | 1.29 | 0.26 | 273.2537 | 273.2544 | -3.09 | 17.60 | C_4_H_8_O_3_ | C_15_H_32_N_2_O_2_ | HMDB00357 | C01089 | Butanoate metabolism |
| 64 | Hexanoic acid | 1.90 | 0.041 | 285.2906 | 285.2909 | -2.94 | 19.22 | C_6_H_12_O_2_ | C_17_H_36_N_2_O | HMDB00535 | C01585 | Fatty acids biosynthesis |
| 65 | Heptanoic acid | 1.48 | 0.082 | 299.3057 | 299.306 | 1.43 | 22.22 | C_7_H_14_O_2_ | C_18_H_38_N_2_O | HMDB00666 | C17714 | Fatty acids biosynthesis |
| 66 | Octanoic acid | 1.69 | 0.095 | 313.3213 | 313.3216 | -0.29 | 25.64 | C_8_H_16_O_2_ | C_19_H_40_N_2_O | HMDB00482 | C06423 | Fatty acids biosynthesis |
| 67 | Octanoic acid isomer | 1.21 | 0.28 | 313.3213 | 313.3222 | -2.02 | 23.06 | C_8_H_16_O_2_ | C_19_H_40_N_2_O | - | - | Fatty acids biosynthesis |
| 68 | Pelargonic acid | 1.37 | 0.18 | 327.3375 | 327.3381 | -3.57 | 28.85 | C_9_H_18_O_2_ | C_20_H_42_N_2_O | HMDB00847 | C01601 | Fatty acids biosynthesis |
| 69 | Decanoic acid | 1.45 | 0.11 | 341.3526 | 341.3533 | -1.52 | 31.55 | C_10_H_20_O_2_ | C_21_H_44_N_2_O | HMDB00511 | C01571 | Fatty acids biosynthesis |
| 70 | Undecanoic acid | 1.43 | 0.26 | 355.3683 | 355.368 | 5.67 | 34.08 | C_11_H_22_O_2_ | C_22_H_46_N_2_O | HMDB00947 | C17715 | Fatty acids biosynthesis |
| 71 | Dodecanoic acid | 1.50 | 0.25 | 369.3839 | 369.3845 | -1.88 | 36.45 | C_12_H_24_O_2_ | C_23_H_48_N_2_O | HMDB00638 | C02679 | Fatty acids biosynthesis |
| 72 | Myristic acid | 1.43 | 0.25 | 397.4152 | 397.4169 | -3.82 | 39.30 | C_14_H_28_O_2_ | C_25_H_52_N_2_O | HMDB00806 | C06424 | Fatty acids biosynthesis |
| 73 | Palmitoleate | 1.31 | 0.52 | 423.4314 | 423.4318 | -2.12 | 39.74 | C_16_H_30_O_2_ | C_27_H_54_N_2_O | HMDB03229 | C08362 | Fatty acids biosynthesis |
| 74 | Palmitic acid | 1.36 | 0.27 | 425.4465 | 425.4501 | -6.73 | 40.23 | C_16_H_32_O_2_ | C_27_H_56_N_2_O | HMDB00220 | C00249 | Fatty acids biosynthesis |
| 75 | Heptadecanoic acid | 1.28 | 0.39 | 439.4627 | 439.4638 | -3.28 | 40.48 | C_17_H_34_O_2_ | C_28_H_58_N_2_O | HMDB02259 | - | Fatty acids biosynthesis |
| 76 | Linolenic acid | 4.93 | 0.026 | 447.4309 | 447.4311 | -0.54 | 39.50 | C_18_H_30_O_2_ | C_29_H_54_N_2_O | HMDB01388 | C06427 | Fatty acids biosynthesis |
| 77 | Linoleic acid | 3.67 | 0.011 | 449.4465 | 449.4468 | -0.75 | 39.99 | C_18_H_32_O_2_ | C_29_H_56_N_2_O | HMDB00673 | C01595 | Fatty acids biosynthesis |
| 78 | Oleic acid | 1.41 | 0.32 | 451.4662 | 451.4627 | -3.43 | 40.36 | C_18_H_34_O_2_ | C_29_H_58_N_2_O | HMDB00207 | C00712 | Fatty acids biosynthesis |
| 79 | Stearic acid | 1.33 | 0.29 | 453.4778 | 453.4806 | -5.03 | 40.66 | C_18_H_36_O_2_ | C_29_H_60_N_2_O | HMDB00827 | C01530 | Fatty acids biosynthesis |
| 80 | Dihomo-γ-linolenic acid | 1.26 | 0.50 | 475.4622 | 475.4599 | 4.42 | 40.68 | C_20_H_34_O_2_ | C_31_H_58_N_2_O | HMDB02925 | C03242 | Fatty acids biosynthesis |
| 81 | Eicosadienoic acid | 2.46 | 0.0078 | 477.4778 | 477.4784 | -0.41 | 40.50 | C_20_H_36_O_2_ | C_31_H_60_N_2_O | - | - | Fatty acids biosynthesis |
| 82 | Eicosenoic acid | 1.53 | 0.18 | 479.4935 | 479.4933 | 0.26 | 40.73 | C_20_H_38_O_2_ | C_31_H_62_N_2_O | - | - | Fatty acids biosynthesis |
| 83 | Eicosanoic acid | 1.36 | 0.30 | 481.5091 | 481.5104 | -2.19 | 41.03 | C_20_H_40_O_2_ | C_31_H_64_N_2_O | HMDB02212 | C06425 | Fatty acids biosynthesis |
| 84 | Docosahexaenoic acid | 3.09 | 0.031 | 497.4465 | 497.4469 | -0.63 | 39.97 | C_22_H_32_O_2_ | C_33_H_56_N_2_O | HMDB02183 | C06429 | Fatty acids biosynthesis |
| 85 | Docosatetraenoic acid | 2.26 | 0.19 | 501.4778 | 501.4778 | 0.73 | 40.39 | C_22_H_36_O_2_ | C_33_H_60_N_2_O | - | - | Fatty acids biosynthesis |
| 86 | Docosadienoic acid | 1.69 | 0.093 | 505.5091 | 505.5084 | 1.04 | 40.83 | C_22_H_40_O_2_ | C_33_H_64_N_2_O | - | - | Fatty acids biosynthesis |
| 87 | Erucic acid | 1.40 | 0.26 | 507.5248 | 507.5249 | -0.37 | 41.08 | C_22_H_42_O_2_ | C_33_H_66_N_2_O | - | - | Fatty acids biosynthesis |
| 88 | Behenic acid | 1.49 | 0.18 | 509.5404 | 509.5413 | -1.35 | 41.74 | C_22_H_44_O_2_ | C_33_H_68_N_2_O | HMDB00944 | C08281 | Fatty acids biosynthesis |
